# 46,XY disorder of sex development and wilms’ tumor due to mutation of WT1 gene: a case report

**DOI:** 10.1186/1687-9856-2015-S1-P113

**Published:** 2015-04-28

**Authors:** Vu Chi Dung, Bui Phuong Thao, Maki Fukami

**Affiliations:** 1 Department of Endocrinology, Metabolism and Genetics, National Hospital of Pediatrics, Hanoi, Vietnam; 2 National Research Institute for Child Health and Development, Department of Molecular Endocrinology, Tokyo, Japan

## 

The Wilms' tumor suppressor gene (WT1) is a transcription factor that plays a major role in development of the gonads and kidneys. It is expressed even earlier than sex-determining region of the Y chromosome in the urogenital ridge from which the gonads and kidneys are derived. WT1 mutations will impair gonadal and urinary tract development and have been demonstrated to cause syndromes of WAGR, Denys–Drash and Fraiser. In this study, our aim is to identify mutation in WT1 gene and to describe clinical features of a Vietnamese patient with 46,XY disorder of sex development (DSD) associated with Wilms’ tumor. DNA was extracted from WBC and mutation analysis of WT1 gene was performed using PCR and direct sequencing. A 5 days newborn presented with penoscrotal hypospadias, microphallus, right testis in the right inguinal and no left testis was found. Karyotype was 46,XY and no ovaries and uterus were found using pelvic ultrasound. Wilms’ tumor was detected at 13 months of age by abdominal ultrasound and CT scan. Mutation analysis was identified a heterozygous missense mutation (c.1390G>A; p.D464N) in exon 9 of WT1 gene. In conclusions, WT1 analysis should be performed in newborns with complex hypospadias with at least one cryptorchid testis and in isolated 46,XY partial to complete gonadal dysgenesis. WT1 analysis is mandatory in all 46,XY DSD with associated kidney disease. Patients with WT1 mutations should be followed up closely because the risk of developing a Wilms' tumor, nephropathy.

Written informed consent was obtained from the patient's parent or guardian for publication of this Case report (and any accompanying images). A copy of the written consent is available for review by the Editor-in-Chief of this journal.

